# Two-Sided Surface Oxidized Cellulose Membranes Modified with PEI: Preparation, Characterization and Application for Dyes Removal

**DOI:** 10.3390/polym9090455

**Published:** 2017-09-16

**Authors:** Wei Wang, Qian Bai, Tao Liang, Huiyu Bai, Xiaoya Liu

**Affiliations:** Key Laboratory of Synthetic and Biological Colloids, Ministry of Education, School of Chemical and Material Engineering, Jiangnan University, Wuxi 214122, China; qianbaichem@163.com (Q.B.); taoliangchem@163.com (T.L.); bhy.chem@163.com (H.B.); lxy@jiangnan.edu.cn (X.L.)

**Keywords:** regenerated celluloses, bioadsorbents, dye removal, functional membranes

## Abstract

Porous regenerated cellulose (RC) membranes were prepared with cotton linter pulp as a raw material. These membranes were first oxidized on both sides by a modified (2,2,6,6-tetramethylpiperidin-1-yl)oxyl (TEMPO) oxidation system using a controlled oxidation reaction technique. Then, the oxidized RC membranes were functionalized with polyethylenimine (PEI) via the glutaraldehyde crosslinking method to obtain bifunctional (carboxyl and amino) porous RC membranes, as revealed by Fourier transform infrared spectroscopy (FT-IR), elemental analysis and zeta potential measurement. The scanning electron microscopy (SEM) and the tests of the mechanical properties and permeability characteristics of modified RC membranes demonstrated that the porous structure and certain mechanical properties could be retained. The adsorption performance of the modified membranes towards dyes was subsequently investigated. The modified membranes displayed good adsorption capacities, rapid adsorption equilibrium and removal efficiencies towards both anionic (xylenol orange (XO)) and cationic (methylene blue (MB)) dyes, making them suitable bioadsorbents for wastewater treatment.

## 1. Introduction

Synthetic dyes are widely utilized in numerous industries (e.g., textile, paper, leather tanning, plastics, rubber, cosmetics and printing) owing to their high stability, relatively low costs and color uniformity characteristics [1,2]. Inevitably, a certain fraction of dyes end up in the effluent during the dyeing process. This colored waste water must be purified before being released into the environment since it is toxic, carcinogen and can damage the aquatic ecological balance [3]. Various methods (e.g., adsorption, membrane separation, chemical oxidation, coagulation-flocculation, photochemical degradation and bioremoval) have been used singly or in combination with other approaches for the removal of dyes [4,5,6]. Among them, adsorption is considered to be economically and environmentally superior to other conventional techniques owing to its easy operation, low cost and effectiveness [7]. However, mechanical agitation is often required to improve the adsorption efficiency by enhancing the contact between the dyes and the adsorbents. Moreover, numerous adsorbents usually require long times before reaching adsorption equilibrium [8].

One paradigmatic adsorbent for the removal of a target pollutant should exhibit a large surface area, good adsorption efficiency and an abundance of adsorption sites while being produced at low cost and in a sustainable and environmentally-friendly manner. Sharma et al. compiled a list of naturally available, low cost and eco-friendly adsorbents for the removal of hazardous dyes from aqueous waste streams via adsorption [4]. Various methods including carbonization, chemical activation and pyrolysis have been proposed to enhance the adsorption efficiency for dyes [9].

Biodegradable and biocompatible cellulose-based materials have been developed in recent decades taking advantage of the wide availability (i.e., the most abundant renewable biopolymer in nature) and low cost characteristics of this polymer. These materials have been used for water treatment purposes especially, as a new class of versatile adsorbents for the removal of dyes. Moreover, the high density of surface hydroxyl groups present on cellulose provides this material with excellent surface modification characteristics, thereby allowing a wide range of functionalization approaches for the adsorption of dyes from aqueous solutions [10]. Thus, different forms of cellulose-based materials have been used as dye adsorbents. Jin et al. prepared amino-functionalized nanocrystalline cellulose, and the sample was then applied as an adsorbent to remove anionic dyes in aqueous solutions [11]. A carboxylate-functionalized adsorbent based on CNCs was prepared, and adsorptive removal of multiple cationic dyes was investigated [12]. Cellulose recycled newspaper fibers were grafted with double quaternary ammonium groups, and the maximum adsorption capacity of this for RTB G-133 was 524 mg·g^−1^ [13]. Luo et al. developed millimeter-scale magnetic regenerated cellulose (RC) beads, and the adsorbent could efficiently adsorb the organic dyes from wastewater, as well as the used adsorbents could be recovered completely [14]. Cellulose nanosponges modified with methyltrioctylammonium chloride were prepared and used for pre-concentration, removal and determination of tartrazine dye, using UV–Vis spectrophotometry [15]. Cellulose powders functionalized with hyperbranched polyethylenimine were prepared, and used for selective dye adsorption and separation based on the unique selective adsorption [16].

Cellulose membranes can be also used as dye adsorbents, benefitting from the porous structure, the adsorption capacity for the removal of positively-charged dyes and good reusable performance, however in the form of multilayer [17], composites [18,19] and cellulose derivatives [20]. However, at present, many studies have focused on using membrane separation technology as the cellulose-based membranes are used for water treatment [21,22], and there are less reports on the pure cellulose functional membranes as adsorbents. Oshima et al., reported phosphorylated bacterial cellulose as an adsorbent for metal Ions [23]. As described above, when used as adsorbents, celluloses need to undergo chemical modification for achieving efficient adsorption. However, during the modification process, celluloses are often subjected to strong acid or oxidant, etc., treatments, so leading to the degradation of cellulose macromolecules [24], which further may damage the porous structures of the cellulose membranes and even destroy membranes themselves. These facts can significantly limit the application of cellulose membranes for adsorption purposes. 

In this study, cotton linter pulp was used as a raw material to prepare porous RC membranes by using a phase-inversion method. Subsequently, the RC membranes underwent a two-sided surface oxidation treatment with a modified (2,2,6,6-tetramethylpiperidin-1-yl)oxyl (TEMPO) oxidation system to yield the corresponding C-6 carboxyl membrane materials. To prevent the cellulose membranes from being damaged via oxidation–degradation of the cellulose macromolecular chains, a control oxidation reaction was carried out by following the method described by Fitz-Binder et al. [25]. Amine groups were subsequently incorporated onto the oxidized cellulose membranes by grafting polyethylenimine (PEI) via the glutaraldehyde crosslinking method [26]. As a result, porous RC membranes containing both carboxyl and amino groups were obtained. Fourier transform infrared spectroscopy (FT-IR), elemental analysis and zeta potential measurement and scanning electron microscopy (SEM) were used to analyze the structure and morphology of the modified cellulose membranes. Furthermore, anionic and cationic dyes were both used as model pollutants to evaluate the dye removal efficiency of the modified cellulose membranes. The adsorption performance and kinetic behaviors during dye adsorption on the cellulose-based adsorbents were further investigated. With the aim to further evaluate the potential application of the modified RC membranes for water treatment purposes, the porosity, pure water permeability characteristics and the mechanical properties of the modified RC membranes were also tested.

## 2. Experimental Section

### 2.1. Materials

Cellulose (cotton linter pulp, α-cellulose ≥95%) was purchased from Hubei Chemical Fiber Group Ltd. (Xiangfan, China). PEI (molecular weight of 600 Da), TEMPO (AR), xylenol orange (XO) (AR) and methylene blue (MB) (AR) were supplied by Aladdin Chemical Reagent Corp., Shanghai, China. Other reagents used in this work were of analytical grade and purchased from Sinopharm Chemical Reagent Co., Shanghai, China.

### 2.2. Preparation of the RC Membranes

RC membranes were prepared according to a reported method [27]. Five grams of cotton linter pulp were added to a LiOH/urea/H_2_O (8.76/12/79.24 in wt %, 100 g) solution, and the resulting aqueous solution was stored at 200 °C for 20 h. The frozen cellulose solution was then vigorously stirred for 5 min at ambient temperature to obtain a transparent cellulose dope. The cellulose dope was then subjected to centrifugation at 8000 rpm for 10 min at −4 °C, and the transparent supernatant fraction was immediately cast on a glass plate. Subsequently, the resulting gel sheets were coagulated with a sulfate aqueous solution to obtain a transparent membrane. The wet membrane was washed with deionized water and dried at ambient temperature to finally obtain the RC membrane.

### 2.3. Preparation of the TEMPO–Oxidized RC Membranes

Oxidized cellulose membranes were prepared according to a reported method [25]. First, the RC membrane was immersed in 1 L of a boric acid (0.1 M) buffer solution (pH = 10.5), and 2 g of NaBr and 0.3 g of TEMPO were subsequently added and mixed by magnetic stirring for 180 min. The pre-wetted membranes were then dried at 60 °C for 5 min. For the printing paste, 5 g of alginate were dissolved in 40 mL of the boric acid buffer solution to form a thickener, and 6 mL of NaOCl were added to the former paste. The mixtures were then stirred to obtain a homogenous paste. Subsequently, the as-prepared thickener paste was applied by printing it on the one-sided surface of the RC membranes containing the NaBr/TEMPO/buffer mixture, and the prints were rested for 60 min. Finally, the printing membrane was thoroughly washed with water. The other side of the cellulose membrane was impregnated in a similar way to generate the two-sided surface TEMPO-oxidized regenerated cellulose (TORC) membrane.

### 2.4. Preparation of Aminated TORC Membranes

The oxidized RC membranes were cut into approximately 80 mm × 70 mm × 0.1 mm pieces and saturated in 50 mL of an anhydrous methanol solution. Four grams of PEI were added, and the resulting solution was stirred at room temperature for 24 h. After that, the wet membranes were rinsed with water to remove the residual PEI and immediately immersed in 100 mL of an anhydrous methanol solution. Subsequently, 20 mL of a glutaraldehyde solution were added dropwise, and the resultant mixtures were stirred at 25 °C for 2 h. Finally, the modified cellulose membranes were repeatedly washed with deionized water to remove the unreacted material and denoted as PEI–TORC membranes.

### 2.5. Characterization of the Functionalized TORC Membranes

The RC and modified RC membranes were chemically characterized by attenuated total reflectance infrared (ATR-IR) spectroscopy (Nicolet 560, Nicolet Co., Ltd., Madison, WI, USA). The spectra were recorded from 600–4000 cm^−1^ with a resolution of 2 cm^−1^ and a minimum of 16 scans. The mass ratios of C, H, O and N of each sample were measured using Elemental Analyzer (Vario EL III, Elementar Co., Langenselbold, Germany). The surface and cross-section morphologies of the RC and modified RC membranes were assessed by using a scanning electron microscope (S-4800, Hitachi Corporation, Tokyo, Japan). Cross-sectional faces of membranes were prepared by being fractured in liquid nitrogen. The samples were deposited on a glass plate and coated with a thin layer of gold/palladium using a sputter coater (K550X, Emitech Ltd., Kent, UK). The pore size analysis of the membrane surface was calculated by using the software of SEM image analysis (Nano Measurer System, Version 1.2.5, Fudan University, Shanghai, China). The surface charge of the PEI-TORC membrane was determined by zeta-potential measurement using an Electrokinetic Analyzer (SurPASS, Anton Paar, Graz, Austria), using a 2 mM KCl electrolyte solution. The tensile strength and strain at break of the membranes were measured on a universal testing machine (Instron 5967, Instron, Norwood, MA, USA), using a 250-N load cell at room temperature. The strain rate was set at 10 mm/min, and five measurements were taken for each sample.

The porosities of the modified membranes were calculated using a reported method [22]. The porosity (*P*) was calculated as Equation (1):

(1) $$P=\frac{(M_{1}-M_{2})/q_{1}}{(M_{1}-M_{2})/q_{1}+M_{2}/q_{2}}\times 100\%$$

The wet membranes were weighed as *M*_1_ and then freeze dried overnight and weighed as *M*_2_. The water content was calculated as *M*_1_ − *M*_2_. *q*_1_ is water density, and *q*_2_ is PEI–TORC density (calculated according to the density of bulk cellulose, 1.5 g cm^−3^).

The water flux of the modified membranes was evaluated using a reported method [28]. The pure water permeability test of the wet membranes was carried out on miniature microfiltration equipment at ambient temperature under an operation pressure of 0.1 MPa, while the water flux was calculated according to Equation (2):

(2) $$J_{w}=\frac{V}{S\times t\times P}$$

where *V* is the volume of solvent passing through the membrane; *t* is the measurement time; *S* is the effective membrane area; *P* is the pressure (0.1 MPa). 

The mean pore radius, *r*_f_, was calculated by employing Equation (3), derived based on the straight-through cylindrical pore model [29].

(3) $$\eta =\sqrt{\frac{8\times \eta \times I\times J}{P\times \Delta P}}$$

where η is the water viscosity (8.9 × 10^−4^ Pa s), *I* is the membrane thickness (m), *J* is the permeation flux (m^3^·m^−2^·s^−1^) and Δ*P* is the load pressure (Pa).

### 2.6. Batch Adsorption Experiments

Batch adsorption experiments were conducted in some 100 mL glass conical flasks in a water bath shaker (25 °C, 200 rpm). Each flask contained 60 mL MB or XO solution and 70 mg adsorbent. The pH of the solution was adjusted by adding 0.1 M HCl or NaOH aqueous solutions. At predetermined time intervals, approximately 3 mL of dye solution were used for UV–Vis measurements and afterwards returned into the flask. This was repeated until equilibrium was reached. The dye removal efficiencies of the RC and PEI–TORC membranes towards XO and MB were investigated for 100 min (dye concentration, 30 mg·L^−1^, pH = 6.8). The effect of pH on the removal efficiency was investigated in the range of 4–11 for 100 min (dye concentration: 30 mg·L^−1^). The effect of initial dye concentration on the adsorption performance was investigated in the range from 30–1230 mg·L^−1^ (at pH values of 6.8 and 4.6) for 120 min. The effect of the contact time (10–100 min) was investigated (dye concentration, 30 mg·L^−1^) at pH 6.8 and 4.6.

The removal efficiency and adsorption capacity of dyes were calculated according to Equations (4) and (5):

(4) $$\mathrm{adsorption}\;\mathrm{capacity}\;(\mathrm{mg}\cdot g^{-1})=\frac{(C_{0}-C_{t})\times V}{m}\times 100$$

(5) $$\mathrm{Removal}\;\mathrm{efficiency}\;(\%)=\frac{C_{0}-C_{t}}{C_{0}}\times 100$$

where *C*_0_ (mg·L^−1^) and *C_t_* (mg·L^−1^) are the initial concentration of the dye and the concentration of the dye at an adsorption time t, respectively, V is the volume of the dye solution and m is the weight of the adsorbent.

The evaluation of the reusability of the adsorbent was carried out at a 0.07 g·L^−1^ dosage of bioadsorbent added into 60 mL of 30 mg·L^−1^ dye solutions for 100 min, and then, the adsorbent was taken out from the solution. The desorption and regeneration of the used adsorbent was performed by immersing the adsorbent in 30 mL of a 0.1 M NaOH or HCl solution for 5 h at room temperature and subsequently washed using distilled water till neutral for the next adsorption. The generated adsorbent was used for another adsorption study in the subsequent cycles.

### 2.7. Adsorption Isotherm and Kinetic Model

The Langmuir and Freundlich models [30] can be used to describe the adsorption dynamic equilibrium process by Equations (6) and (7):

(6) $$\frac{c_{e}}{q_{e}}=\frac{1}{k_{1}q_{\max}}+\frac{c_{e}}{q_{\max}}$$

(7) $$Inq_{e}=Ink_{f}+\frac{Inc_{e}}{n}$$

where *q*_e_ (mg·g^−1^) is the equilibrium adsorption capacity, *q*_max_ (mg·g^−1^) is the maximum adsorption capacity, *C*_e_ (mg·L^−1^) is the equilibrium concentration of free dye molecules in the solution (mg·L^−1^), *K*_1_ is the Langmuir constant and *K*_f_ and 1/*n* are Freundlich constants.

In order to explore the adsorption mechanism of the rate limiting steps involved, the pseudo-first order and the pseudo-second order kinetic models [31] were used to study the type of adsorption and the adsorption mechanism. The first and second order rate equations can be expressed as Equations (8) and (9):

(8) $$\log(q_{e}-q_{t})=\log q_{e}-\frac{k_{1}t}{2.303}$$

(9) $$\frac{t}{q_{t}}=\frac{1}{k_{2}q_{e}{}^{2}}+\frac{t}{q_{e}}$$

where *q*_e_ and *q*_t_ are the amounts of adsorbed dye per unit mass of adsorbent (mg·g^−1^) at equilibrium and a time *t*, respectively, and *k*_1_ and *k*_2_ are the first order and the pseudo-second order adsorption rate constants, respectively.

## 3. Results and Discussion

### 3.1. Characterization of the Modified RC Membranes

**Figure 1:**
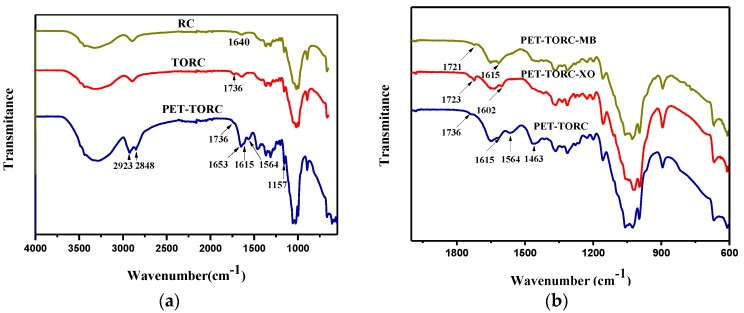
(**a**) FTIR spectra of RC, TORC, polyethylenimine(PEI)-(2,2,6,6-tetramethylpiperidin-1-yl)oxyl (TEMPO)-oxidized regenerated cellulose (TORC); (**b**) FTIR spectra of xylenol orange (XO)-adsorbed PEI–TORC and MB-adsorbed PEI–TORC.

The FTIR spectra of the RC, TORC and PEI-TORC membranes are shown in Figure 1a. Compared with the unmodified RC, the FTIR spectra of the TORC membranes showed a new peak at 1736 cm^−1^, which was attributed to the C=O stretching frequency of the carboxyl group [32], thereby revealing a successful TEMPO-oxidation process. In the case of the PEI-TORC membranes, new absorption bands were observed at ca. 1653 cm^−1^ corresponding to the C=N stretching vibration, which was formed in the glutaraldehyde crosslinking process. Three new peaks appeared in the 1600–1800-cm^−1^ region and were ascribed to the C=O stretching vibration of carboxyl groups (1736 cm^−1^), the N–H bending vibration of secondary (1615 cm^−1^) and primary (1564 cm^−1^) amines [33,34]. Furthermore, the large enhancement of the C–C skeleton vibration at 1157 cm^−1^ and the presence of the C–H characteristic peaks at 2924 and 2849 cm^−1^ for the PEI-TORC membranes further demonstrated the successful crosslinking reaction.

In order to evaluate the change of the content of the elements in the whole material after modification, the contents of C, N, O and H in different cellulose-based membranes are listed in Table 1. After TEMPO oxidation, the oxygen content in cellulose increased from 49.11–51.29%. The nitrogen content of PEI-TORC increased significantly from 0–3.87% compared to that of TORC. The elemental analysis data and the FTIR results both confirmed the successful oxidation of cellulose and PEI grafting on TORC.

FT-IR is a useful tool to study the possible adsorbent-adsorbate interactions. The FT-IR spectra of the cellulose-based and the dye-loaded bioadsorbents are shown in Figure 1b. After dye adsorption, the adsorption peaks of the bioadsorbent shifted towards lower wavenumbers. For example, the adsorption peak attributed to the C=O stretching vibration shifted from 1736 to 1725 cm^−1^ (for XO) and 1724 cm^−1^ (for MB). The absorption bands assigned to the N–H stretching vibration shifted from 1615 to 1602 cm^−1^ (for XO) and 1613 cm^−1^ (for MB). After absorption, the disappearance of the N–H bending vibration peak of primary amines (1564 cm^−1^) and the variation of the CH_2_ bending peak (1463 cm^−1^) also revealed the existence of electrostatic and hydrogen bonding interactions between the functional groups of the adsorbent and the dye molecules.

**Figure 2:**
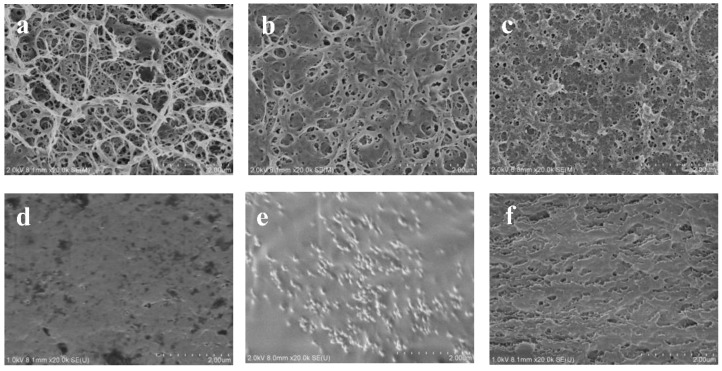
SEM images of the RC membrane: (**a**) surface, (**d**) cross-section; TORC membrane: (**b**) surface, (**e**) cross-section; PEI-TORC membrane: (**c**) surface, (**f**) cross-section.

The SEM images for the RC, TORC and PEI-TORC membranes are shown in Figure 2. Surface morphology changes of cellulose-based membranes were observed; the porous structure of RC was maintained after TEMPO oxidation and crosslinking reaction. The average diameter of the pores of the pure cellulose membrane surface from SEM (78 nm, standard deviation of 18) was larger than that of PEI-TORC (43 nm, standard deviation of 10), revealing the reaction on the surface of the cellulose, introducing new groups and molecular chains on the membrane surface. As shown in the SEM images of the cross-section of cellulose-based membranes, compared with the cross-section morphology of RC membranes, the interior structures of TORC and PEI-TORC membranes had changed greatly. The cross-section of the RC membrane was relatively smooth. Interestingly, it can be observed that the cross-section image of the TORC membrane shows a non-homogeneous structure. This is because the control oxidation reaction leads to the fact that the pore structure and the microstructure of the material are different between the surface and the interior of the TORC membrane. When fractured in liquid nitrogen, there is a different morphology from the homogeneous RC membrane. Compared with the cross-section morphology of PET-TORC membranes, the cross-section of the PEI–TORC membrane became flat, suggesting that the uniformity of the membrane structure was improved due to the crosslinking reaction; however, the pore size and shape of the middle part of the membrane are different from those of the peripheral part, suggesting both oxidation and crosslinking reaction affected the morphology and structure of the PEI-TORC membranes. Moreover, this structural feature makes it unsuitable as a molecular sieve. 

**Figure 3:**
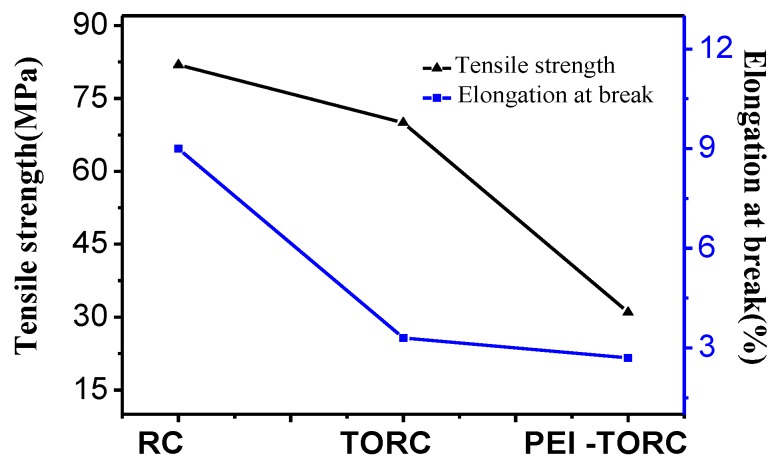
Tensile strength and elongation at break of RC, TORC and PEI-TORC membranes.

As shown in Figure 3, the mechanical properties of the modified membranes were reduced. The tensile strength and elongation at break of RC membranes were 81.9 MPa, and 9.0%, respectively, while the tensile strength and elongation at break of PEI–TORC membranes were 31.0 MPa and 2.7%, respectively. The decrease of the mechanical properties of TORC membranes was due to the oxidative degradation of cellulose and the non-uniformity of the membrane materials caused by the control of the oxidation reaction. The decrease in elongation at break of the PEI-TORC membranes was due to the fact that the crosslinking reaction limits the movement of the molecular chain, and the descent in the tensile strength is likely due to the inhomogeneity of the material. 

In order to explore the potential application value of membrane in water treatment, the structure and performance of the PEI–TORC membrane were further studied. The porosity, water flux and pore size of the PEI–TORC membrane were 72%, 6.14 L·m^−2^·h^−1^·bar^−1^ and 25.9 nm (the mean pore size), respectively. Importantly, it is possible to adjust the morphology, structure and properties of the PEI–TORC membrane by changing the various influencing factors in the preparation process, making it possible to further expand the application range of the modified cellulose membrane.

### 3.2. Adsorption Properties of the RC and PEI-TORC Membranes

**Figure 4:**
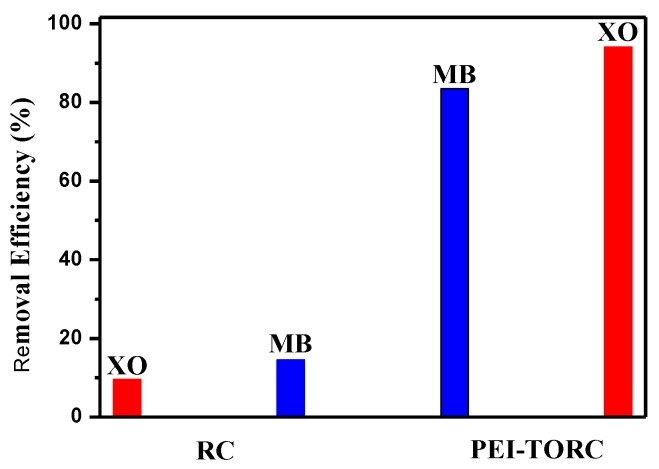
The removal efficiencies of RC and PEI-TORC (initial dye concentration: 30 mg·L^−1^, 60 mL MB or XO solution, 70 mg adsorbent, 100 min, pH = 6.8).

The dye removal percentages of the RC and PEI–TORC membranes are also shown in Figure 4. RC membranes exhibited a poor adsorption capacity, meaning that raw cellulose did not have enough binding sites for dye adsorption. However, the adsorption capacity of hydroxyl for the removal of positively-charged dyes, the hydroxyl groups on the surface, is quite limited because most of them are included in the intra- and inter-molecule hydrogen-bond network. The removal efficiency of the PEI-TORC membrane was significantly higher than that of the RC material. The sorption of dyes onto adsorbents may well include chemical sorption, which could greatly improve the adsorption capacity. From the change in the FTIR of the functions in PEI–TORC upon the adsorption of MB or XO, it is apparent that the adsorption process is likely to involve chemical sorption.

### 3.3. Effect of the pH on Adsorption

**Figure 5:**
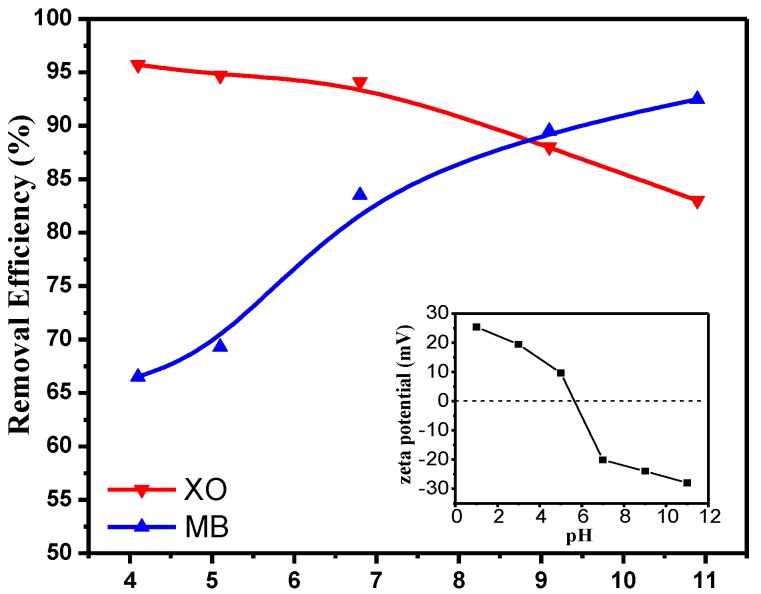
Effect of pH on the dye removal efficiencies of XO and MB (initial dye concentration: 200 mg·L^−^^1^, 30 mL dye solution, 70 mg adsorbent).

The solution pH significantly alters the level of electrostatic or molecular interaction between the adsorbent and the adsorbate due to the charge distribution on the material [11]. Thus, the pH of the solution is one of the determinants of the efficiency of the adsorbent for dye removal, as shown in Figure 5. The zeta potentials of PEI–TORC at various pH are also shown in Figure 5. The results showed that PEI–TORC exhibited a positively-charged surface at a pH lower than 5.7, while a negatively-charged surface at a pH higher than 5.7, which is shown to be electrically neutral (i.e., zero point charge). Xylenol orange is a negatively-charged species [35], while the methylene blue molecules are positively charged [36]. PEI–TORC exhibited positive surface charge at a pH lower than 5.7, leading to electrostatic attraction between the bioadsorbent and the anionic group of XO, resulting in maximum removal efficiency of 95.7% at pH 4.1. However, the PEI–TORC exhibited a negatively-charged surface, resulting in weaker electrostatic interactions between the bioadsorbent and XO and lower dye adsorption [37], with increasing the pH to higher than 5.7. The efficiency of the bioadsorbent for MB dye removal kept increasing in the pH range of 4.1–10.9, indicating the electronegativity of PEI–TORC continued to increase with increasing pH values. The results revealed the excellent dye removal efficiency of the bioadsorbent for both anionic and cationic dyes. The high adsorption of cationic or acidic dyes at higher pH may be due to the unique molecular structure of this bioadsorbent. However, considering the practical application and the zeta potential of the adsorbent, this paper mainly studied the adsorption performance of the bioadsorbent at pH 4.6 and 6.8.

### 3.4. Effect of the Initial Dye Concentration and the Adsorption Isotherm

**Figure 6:**
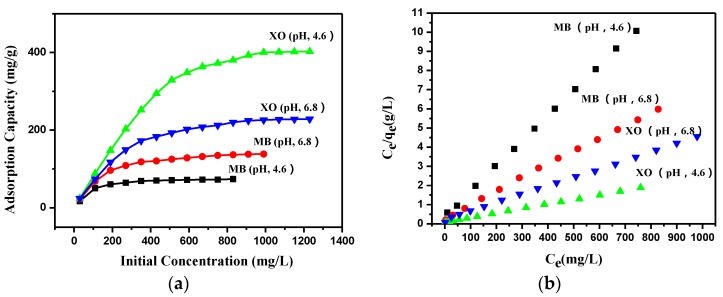
(**a**) Effect of initial concentration on dye adsorption by PEI-TORC membranes; (**b**) the plots of *C*_e_/*q*_e_ against *C*_e_ for adsorption of XO and MB at different pH.

As shown in Figure 6a, the dye adsorption capacity of PEI-TORC increased with the increase of the initial concentration and then tended to level off, resulting from the increasing driving force from the concentration gradient [38]. However, the equilibrium adsorption capacity and the adsorption behavior of PET–TORC varied greatly due to different types of dyes and different pH values. The adsorption capacity of XO was higher than that of MB, which was attributed to the limited number of carboxyl groups on the surface of the bioadsorbent, caused by controlled oxidation. The maximum adsorption capacities of XO and MB reached 403 and 74 mg·g^−1^, at pH 4.6, respectively; while the maximum values of XO and MB reached 229 and 139 mg·g^−1^, at pH 6.8, respectively.

The adsorption isotherm study was carried out on two well-known isotherms, Langmuir and Freundlich. When *C*_e_/*q*_e_ was plotted against *C*_e_, a straight line with slope 1/*q*_max_ was obtained (Figure 6b), indicating that the adsorption of the both dyes onto PEI–TORC follows the Langmuir isotherm. The *q*_max_ values of the Langmuir model for adsorption of MB were 77 mg·g^−1^ (pH, 4.6) and 144 mg·g^−1^ (pH, 6.8), while the *q*_max_ values for adsorption of XO were 420 mg·g^−1^ (pH, 4.6) and 241 mg·g^−1^ (pH, 6.8), which was similar to the experimental *q*_max_ values obtained from Figure 6a. Thus, these results indicated that the PEI-TORC membrane exhibited relatively high effectiveness in removing the tested dyes, especially anionic dyes. Moreover, the isotherm parameters are summarized in Table 2. It is evident that the Langmuir isotherm model fits the experimental data better than the Freundlich model. 

### 3.5. Effect of the Contact Time and the Adsorption Kinetics

**Figure 7:**
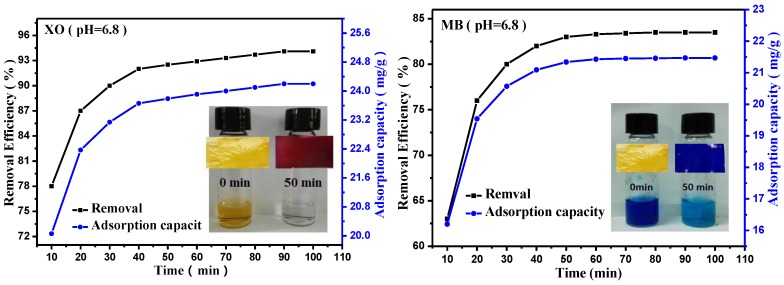

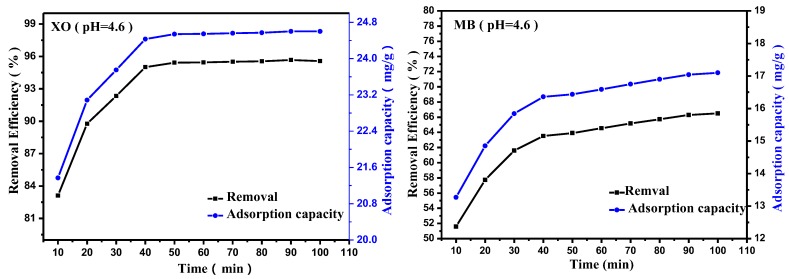
Effect of contact time on the dye removal efficiencies of XO and MB (initial dye concentration: 30 mg L^−1^,60 mL MB or XO solution, 70 mg adsorbent, 10–100 min, pH = 6.8 or 4.6).

Figure 7 shows the dye removal efficiency and color change of XO and MB as a function of the adsorption time. The percentage of dye removal increased rapidly within the first 40 min, and the absorption equilibrium was reached at ca. 100 min. Furthermore, the color of the XO and MB solutions gradually became lighter with the adsorption time. The color of the XO solution completely disappeared after the adsorption process. Accordingly, the PEI–TORC membrane shifted from dark red to orange after adsorption, in line with the dye removal efficiency results. In the adsorption stage, the large number of hydrophilic hydroxyl, carboxyl and amino groups on the surface of the adsorbent resulted in electrostatic interactions that occurred upon rapid migration of the anionic XO or cationic MB dyes to the surface adsorption sites (i.e., –COOH and –NH_2_) on the bioadsorbent, and the number of dye molecules adsorbed on the membrane increased with the adsorption time and levelled off at 50 min as a result of electrostatic repulsion forces between the adsorbed dye molecules. Therefore, the optimum contact time for the adsorption of XO and MB was ca. 50 min (i.e., 93% of XO and 83% of MB were removed). These results demonstrated that the PEI-TORC membrane is a good adsorbent for the rapid removal of XO and MB from waste waters.

As shown in Table 3, the *R*^2^ values for MB and XO indicated that pseudo-second order (PSO) kinetic models could be used to predict the behavior over the whole range of the adsorption process, which indicating that the intraparticle diffusion was involved in the adsorption process. At a pH of 6.8, the *q*_e_ values for XO and MB calculated with a pseudo-second order model were 25 and 22 mg·g^−1^, respectively, and these values changed to 25 and 18 mg·g^−1^ at a pH of 4.6. These results were in good agreement with the experimental data.

### 3.6. Reusability 

**Figure 8:**
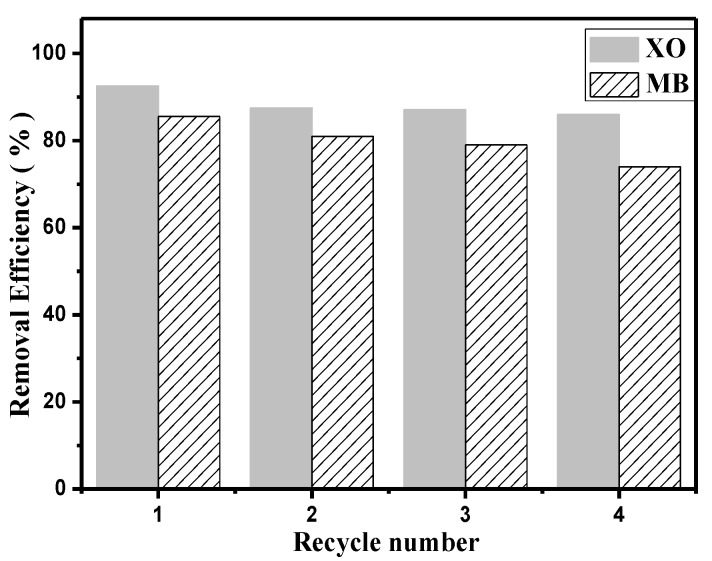
The dye removal efficiency of XO and MB after three desorption-adsorption cycles.

Figure 8 shows the reusability of the PEI–TORC membrane. After three consecutive desorption-adsorption cycles, the dye removal rates towards XO and MB decreased by 6 and 11%, respectively. Therefore, the adsorbent showed high reusability for the removal of XO and MB.

## 4. Conclusions 

Functional membranes based on regenerated cellulose were prepared by grafting PEI onto controlled oxidized RC membranes. Modification conditions were screened to achieve the biofunctionalization of the regenerated cellulose membranes successfully and to retain the porous membrane structure. The maximum adsorption capacities of cationic and anionic dyes was observed, being the highest for xylenol orange (403 mg·g^−1^), followed by methylene blue (139 mg·g^−1^). Furthermore, the membranes showed low flux, supporting the usefulness of them as adsorbents. One may adjust the morphology, structure and properties of the PEI-TORC membrane by changing the various influencing factors in the preparation process, for exploring its potential application in the future.

## Figures and Tables

**Table 1:** Elements analysis results of unmodified and modified cellulose membranes.

Sample	C (%)	O (%)	H (%)	N (%)
RC	41.02	49.11	9.87	0
TORC	39.64	51.29	9.07	0
PEI-TORC	41.98	45.91	8.24	3.87

**Table 2:** Isotherm parameters for the adsorption of XO and MB onto PEI–TORC.

	Langmuir Model				Freundlich Model		
pH	Dyes	*K*_l_ (L·mg^−1^)	*q*_max_ (mg·g^−1^)	*R*^2^	*n*	*K*_f_ (mg·g^−1^)	*R*^2^
6.8	XO	0.018	240.96	0.9981	2.89	25.28	0.9465
	MB	0.024	144.09	0.9987	3.12	18.64	0.8902
4.6	XO	0.030	420.17	0.9995	2.56	39.30	0.8922
	MB	0.031	76.57	0.9998	3.36	11.90	0.8170

**Table 3:** Kinetic parameters for the adsorption of XO and MB onto PEI–TORC.

	Pseudo-First Order Model				Pseudo-Second Order Model		
pH	Dyes	*K*_1_ (g·mg^−1^ min^−1^)	*q*_e_ (mg·g^−1^)	*R*^2^	*K*_2_ (g·mg^−1^·min^−1^)	*q*_e_ (mg·g^−1^)	*R*^2^
6.8	XO	0.049	5.16	0.9753	0.0092	24.75	0.9999
	MB	0.092	13.01	0.9954	0.017	22.16	0.9996
4.6	XO	0.072	5.09	0.9144	0.028	25.03	0.9999
	MB	0.054	7.61	0.9631	0.014	17.84	0.9997
